# Relation between the Global Burden of Disease and Randomized Clinical Trials Conducted in Latin America Published in the Five Leading Medical Journals

**DOI:** 10.1371/journal.pone.0001696

**Published:** 2008-02-27

**Authors:** Pablo Perel, J. Jaime Miranda, Zulma Ortiz, Juan Pablo Casas

**Affiliations:** 1 Department of Epidemiology and Population Health, London School of Hygiene and Tropical Medicine, London, United Kingdom; 2 School of Public Health and Administration, Universidad Peruana Cayetano Heredia, Lima, Peru; 3 Epidemiological Research Institute, National Academy of Medicine, Buenos Aires, Argentina; Public Library of Science, Canada

## Abstract

**Background:**

Since 1990 non communicable diseases and injuries account for the majority of death and disability-adjusted life years in Latin America. We analyzed the relationship between the global burden of disease and Randomized Clinical Trials (RCTs) conducted in Latin America that were published in the five leading medical journals.

**Methodology/Principal Findings:**

We included all RCTs in humans, exclusively conducted in Latin American countries, and published in any of the following journals: *Annals of Internal Medicine*, *British Medical Journal*, *Journal of the American Medical Association*, *Lancet*, and *New England Journal of Medicine*. We described the trials and reported the number of RCTs according to the main categories of the global burden of disease. Sixty-six RCTs were identified. Communicable diseases accounted for 38 (57%) reports. Maternal, perinatal, and nutritional conditions accounted for 19 (29%) trials. Non-communicable diseases represent 48% of the global burden of disease but only 14% of reported trials. No trial addressed injuries despite its 18% contribution to the burden of disease in 2000.

**Conclusions/Significance:**

A poor correlation between the burden of disease and RCTs publications was found. Non communicable diseases and injuries account for up to two thirds of the burden of disease in Latin America but these topics are seldom addressed in published RCTs in the selected sample of journals. Funding bodies of health research and editors should be aware of the increasing burden of non communicable diseases and injuries occurring in Latin America to ensure that this growing epidemic is not neglected in the research agenda and not affected by publication bias.

## Introduction

It has been estimated that less than 10% of health research spending is directed toward diseases or conditions that account for 90% of the global burden of disease, a phenomenon referred to as the “10/90 gap”.[1] The gap is also reflected in the low proportion of publications arising from research in low and middle income countries, where most of the global burden of disease occurs.[2]

Addressing context specific research questions are fundamental to designing interventions that improve health. Randomized controlled trials (RCTs) are the gold standard to assess the effectiveness of interventions. An unbiased design and adequate reporting of RCTs that specifically address diseases which affect low and middle income countries can have an impact in changing medical practice and influencing public health policy.

Five of the world's leading medical journals—Annals of Internal Medicine, British Medical Journal (BMJ), Journal of the American Medical Association (JAMA), Lancet, and New England Journal of Medicine (NEJM)—have together the highest impact in medical research, and therefore, they can have a large influence on clinical medicine and public health worldwide. In relation to this group of medical journals, one of its editors stated that “Their international success brings global responsibilities to the communities they serve and profit from…” and that they should raise “the priority of papers from less-developed countries in line with global burdens of disease”.[3] Unfortunately, there is good evidence of the under representation of research addressing common conditions from developing countries in these leading journals.[2], [4], [5], [6]

Bibliometric analyses—a set of methods used to study or measure texts and information—have been done to illustrate the gaps of the published medical literature, either in terms of subjects covered, representation of journal’s editorial boards, and specifically, the under-representation of low and middle income countries in high-impact general medical journals.[6], [7], [8], [9]

Latin-American and Caribbean countries have experienced a rapid epidemiological transition and since 1990 non communicable diseases and injuries already account for the majority of death and disability-adjusted life years in the region.[10], [11] No previous studies have investigated the relationship between RCTs and the burden of disease in Latin America. Description of this relationship can provide information about gaps between the health needs and the research conducted in the region. This information can contribute to the establishment of a research agenda and prioritize neglected conditions.

We analyzed the relationship between the global burden of disease and RCTs conducted in Latin America published in five leading medical journals.

## Methods

### Eligibility Criteria

We included all RCT s in humans, exclusively conducted in Latin American countries (by this we mean that the population was located in Latin America irrespective of the origin of the researcher) and published in any of the following journals: *Annals of Internal Medicine*, *BMJ*, *JAMA*, *Lancet* and *NEJM*. The definition used for RCTs was an experiment in which investigators randomly allocate eligible participants into an intervention group (arm) each of which receives one or more of the interventions (or none) that are being compared.[12]

### Identification of the trials

We searched the PubMed database registers from 1990 up to December 2006 using the *Cochrane Sensitive Search Strategy*, which is highly sensitivity to identify RCTs, and combined it with the terms “Latin America” or “Latin America” and all the country names from the region.[13] We limited the search strategy to the five selected medical journals (*Annals of Internal Medicine*, *BMJ*, *JAMA*, *Lancet* and *NEJM*). (See Appendix S1 for the full search strategy).

### Data extraction

From selected trials, the following data were extracted: name of the first author, year of publication, country(ies) where the study was conducted, number of participants, description of disease evaluated according to groups of global burden of disease and type of funding. Data extraction and data entry was made independently by two reviewers. Differences in data extraction were resolved by a third party.

### Data analysis

We conducted a descriptive analysis listing the number of RCTs, participants and countries in which the studies were conducted. We also reported the number of RCTs according to the main categories of the global burden of disease.

## Results

Of 181 reports initially obtained, 66 (36%) fulfilled the inclusion criteria and were retrieved for the analysis.

Table 1 displays the general characteristics of the reports included. The majority of the trials were published in *Lancet* and the countries with the highest numbers of publications were Brazil and Mexico. Cluster trials accounted for one-fifth of all the publications. We found no defined trend in the frequency of publications during this time. Most of the trials were non-commercial.

**Table 1:** General Characteristics of the reports included

Publications per journal	n (%)
Lancet	30 (46)
BMJ	12 (18)
NEJM	12(18)
JAMA	6 (9)
Annals	6 (9)
Publications per country*	n
Brazil	14
Mexico	12
Argentina	9
Chile	6
Venezuela	5
Haiti	5
Peru	5
Colombia	4
Unit of randomization	n (%)
Individuals	54 (81)
Clusters	12 (19)
Date of publication	n (%)
1990–1992	10 (15)
1993–1995	14 (22)
1996–1998	10 (15)
1999–2001	8 (12)
2002–2004	12 (18)
2005–2006	12 (18)
Type of funding	n(%)
Non commercial	42 (64)
Commercial	6 (9)
Both	12 (18)
Not reported	6(9)
Sample Size	mean (range)
Patients included per report	275 (26 to 348,139)
Conditions according to the Global Burden of Disease Study	n (%)
Communicable diseases	38 (57)
Maternal perinatal and nutritional	19 (29)
Non-Communicable diseases	9 (14)

Communicable diseases accounted for 38 (57%) reports. Maternal, perinatal, and nutritional conditions accounted for 19 (29%) trials, and 9 (14%) addressed non-communicable diseases. Within the latter group, 4 trials evaluated cardiovascular disease, 1 cancer and 4 other non-communicable diseases. We found no RCTs assessing interventions for injuries. The most common individual condition evaluated was traveler’s diarrhea, which was studied in 4 (6%) of the trials. (See table 2 for the full details of reports included).

**Table 2:** Full details of the included reports

Year	Journal	Participants	Cluster	Funding	Specific Condition	Global Burden of Disease Classification	Countries
1990	Lancet	81621	no	Non-commercial	Salmonela Tiphy	Communicable	Chile
1990	Jama	227	no	Non-commercial	traveller’s diahrrea	Communicable	Mexico
1991	NEJM	101	no	Commercial	Infant meningitis	Communicable	Costa Rica
1991	NEJM	1194	no	Non-commercial	Hipertensive disorders in pregnancy	Maternal, perinatal and nutrition	Argentina
1991	NEJM	86	no	Commercial	Infant diarrea	Maternal, perinatal and nutrition	Costa Rica
1992	NEJM	2235	no	Non-commercial	Delivery and low birth weight	Maternal, perinatal and nutrition	Argentina, Brasil, Cuba, and Mexico
1992	Jama	191	no	Both	traveller’s diahrrea	Communicable	Mexico
1992	NEJM	110	no	Not reported	Leshmaniasis	Communicable	Colombia
1992	Lancet	29113	no	Non-commercial	Lepra	Communicable	Venezuela
1992	Jama	278	no	Commercial	DTP vaccine response	Communicable	Chile
1993	Lancet	2606	no	Non-commercial	Delivery, episiotomy	Maternal, perinatal and nutrition	Argentina
1993	Annals	126	no	Commercial	Artritis reumatoidea	NCD	Mexico
1993	Lancet	11124	si	Both	Diarrhea and respiratory infections in infants	Communicable	Haiti
1993	Lancet	118	no	Non-commercial	HiV and Tuberculosis	Communicable	Haiti
1993	NEJM	275	no	Both	Diarrea	Communicable	Peru
1993	Lancet	1548	no	Non-commercial	Malaria	Communicable	Colombia
1993	Lancet	159	no	Non-commercial	Infant diarrea	Communicable	Guatemala and Brazil
1993	Lancet	4534	no	Commercial	Acute Miocardial Infarction	NCD	Argentina, Brasil, Chile, Paraguay, Uruguay and Venezuela
1994	Lancet	88	no	Non-commercial	traveller’s diahrrea	Communicable	Belize
1994	Lancet	1563	no	Not reported	Colera	Communicable	Peru
1994	Lancet	275	no	Non-commercial	Kangaroo low birth weight infants	Maternal, perinatal and nutrition	Ecuador
1994	Lancet	516	no	Not reported	Heart Failure	NCD	Argentina
1994	Lancet	141	no	Non-commercial	Infant nutrition	Maternal, perinatal and nutrition	Honduras
1994	Lancet	1240	no	Non-commercial	Diarrea e infecciones respitatorias pediatricas	Maternal, perinatal and nutrition	Brasil
1996	Lancet	130	no	Non-commercial	Chagas	Communicable	Brasil
1997	NEJM	2207	no	Both	Rotavirus Diarrrea	Communicable	Venezuela
1997	BMJ	472	no	Non-commercial	Childhood Pneumonia	Communicable	Brasil
1997	Lancet	113	no	Non-commercial	Filariasis in childrem	Communicable	Haiti
1997	Annals	187	no	Not reported	Leshmaniasis	Communicable	Colombia
1997	Lancet	202	no	Both	Coronary Heart Disease	NCD	Argentina
1997	BMJ	591	no	Non-commercial	Blood pressure of children	Maternal, perinatal and nutrition	Argentina
1998	Annals	176	no	Non-commercial	Malaria	Communicable	Colombia
1998	Lancet	627	no	Non-commercial	Haemofilus in infants	Communicable	Chile
1998	NEJM	53	no	Not reported	Acute Respiratory Syndrome	Communicable	Brasil
1999	BMJ	101	no	Both	Snake bite	Communicable	Brasil
1999	Jama	543	no	Non-commercial	Meningitis	Communicable	Chile
1999	Lancet	130	no	Non-commercial	Breastfeeding	Maternal, perinatal and nutrition	Mexico
2000	Lancet	233	no	Non-commercial	Tuberculosis	Communicable	Haiti
2000	Lancet	12926	no	Non-commercial	HIV	Communicable	Nicaragua
2000	Annals	42	no	Non-commercial	HIV	Communicable	Haiti
2000	NEJM	135	no	Commercial	Infant diarrea	Communicable	Peru
2001	Jama	1193	no	Both	Respiratory tract infection in infants	Communicable	Dominicana
2002	BMJ	2913	si	Both	Leshmaniasis	Communicable	Venezuela
2002	Lancet	26	no	Non-commercial	Altitude polycythaemia	NCD	Bolivia
2003	BMJ	301	no	Non-commercial	Agitation in mental health	Maternal, perinatal and nutrition	Brasil
2003	Lancet	240	no	Non-commercial	Depression	Maternal, perinatal and nutrition	Chile
2004	Lancet	70 clusters	si	Non-commercial	Infant and maternal health	Maternal, perinatal and nutrition	Honduras
2004	BMJ	210	si	Non-commercial	Snake bite	Communicable	Ecuador
2004	BMJ	139	si	Non-commercial	Infant malnutrition	Maternal, perinatal and nutrition	Jamaica
2004	Lancet	149276	si	Non-commercial	Delivery	Maternal, perinatal and nutrition	Argentina, Brasil, Cuba, Guatemala and Mexico
2004	Jama	795	si	Non-commercial	Infant anaemia and nutrition	Maternal, perinatal and nutrition	Mexico
2004	BMJ	120	no	Non-commercial	Tetanus	Communicable	Brasil
2004	NEJM	68	no	Not reported	Cardia arrest	NCD	Brasil
2004	NEJM	120	no	Both	Neurocisticercosys	Communicable	Peru
2005	Lancet	348139	si	Non-commercial	Tuberculosis	Communicable	Brasil
2005	Lancet	350	no	Non-commercial	Breastfeeding	Maternal, perinatal and nutrition	Brasil
2005	BMJ	1518	no	Non-commercial	Heart Failure	NCD	Argentina
2005	Lancet	277	si	Non-commercial	Infan nutrition	Maternal, perinatal and nutrition	Peru
2005	Annals	210	no	Both	traveller’s diahrrea	Communicable	Mexico
2005	NEJM	162	no	Both	Lupus	NCD	Mexico
2006	BMJ	232	no	Non-commercial	Depression	Maternal, perinatal and nutrition	Jamaica
2006	Lancet	476	no	Non-commercial	Infant anaemia	Maternal, perinatal and nutrition	Mexico
2006	BMJ	10049	si	Both	Dengue	Communicable	Venezuela
2006	Lancet	2373	si	Non-commercial	Parasites	Communicable	Ecuador
2006	BMJ	10954	si	Non-commercial	HIV	Communicable	Mexico
2006	Annals	30	no	Non-commercial	Glioblastoma	NCD	Mexico

**Figure 1:**
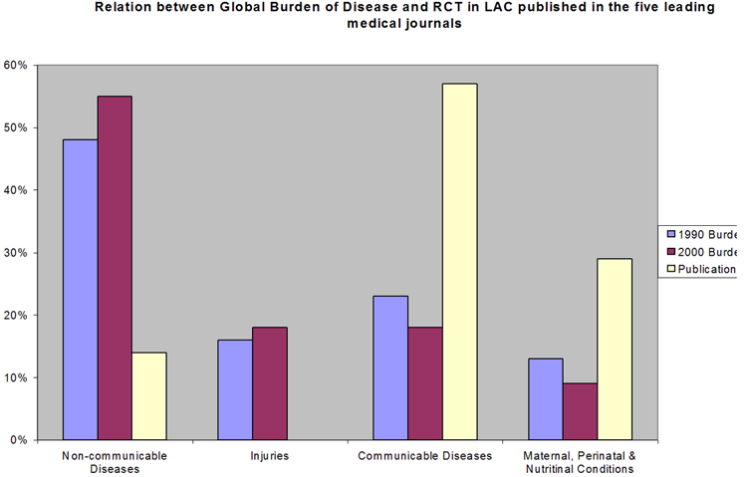
Relation between Global Burden of Disease and RCTs in LAC published in the five leading medical journals. The figure shows an evident mismatch: non-communicable diseases represented 48% of the burden of disease in LAC and 14% of the RCTs. No RCTs addressed injuries despite these representing 18% of the DALYs in 2000.

We found an overall poor correlation between the global burden of disease and the topic of the published RCTs conducted in Latin America. The mismatch is evident in the following figures: non-communicable diseases represent 48% of the burden of disease and 14% of the RCTs; no RCTs addressed injuries despite these representing 18% of the burden of disease in 2000 (see Figure 1).

## Discussion

There is an evident mismatch between the burden of disease in and publications from Latin America. Non communicable diseases and injuries account for up to two thirds of the burden of disease in Latin America but these topics are seldom addressed in published RCTs in the selected sample of journals. Most of the reports that were retrieved evaluated infectious diseases, and within this group, the predominant condition studied was traveler’s diarrhea, a condition that may be more relevant to travelers from developed countries visiting less developed areas. Previous studies have reported the paucity of RCTs addressing the diseases of low and middle income countries.[5], [14], [15] However, to the best of our knowledge previous studies have not explored the relation between global burden of disease and RCTs in Latin America.

The gap identified in our study may have different explanations including lack of original research that mirror the burden of disease or a higher rate of RCTs publications addressing communicable, maternal, perinatal and nutritional conditions. The latter could be due to better quality of the design and reporting of the original studies. Alternatively editors and reviewers from the leading journals could be biased to include publications from Latin America when they focus on these conditions.

### Why is important to conduct RCTs in Latin America?

As previously stated, context is crucial to decide which interventions are effective in specific populations because the effect—and therefore the impact—of some interventions could differ according to the setting. This is of particular relevance for those interventions that address behavioral modifications or involve health services.[16] Preventive interventions in injuries targeting behaviors that could be strongly influenced by cultural conditions or the adherence to medication in chronic conditions like cardiovascular diseases are some of the situations where RCTs are needed. A good example of this type of research is the DIAL Trial which addressed the case management of patients with chronic heart failure.[17] Furthermore, for chronic diseases the relevant outcome of interest is quality of life, so local evidence is necessary since this type of “soft” outcome is more culturally dependent. Another important reason to conduct trials in the region is that certain diseases predominantly occur in Latin America, such as Chagas disease[18] or Hemorrhagic Argentine Fever. These conditions may be of more interest to the region’s population and scientific community.

### Why is important to publish the results of the RCTs?

A fundamental step after study completion is to publish the findings in scientific journals. The publication in the five leading medical journals, in particular, can influence clinical practice and policy.[19] These journals are particularly relevant in low and middle income where limited resources require that medical libraries subscribe to only a few international journals, and therefore, prioritize those with the higher impact. The publication of RCTs conducted on the region will not only disseminate the results appropriately but will also raise the awareness of the topic and potentially could help to increase the funding for research in these conditions.

### Limitations

A limitation of our study is that we only considered five leading medical journals. We acknowledge that many of the trials conducted in Latin America may have been published in different journals, however we considered that those published on the leading journals will have the larger visibility and impact amongst the medical and health care community.

Another important limitation is that we included RCTs exclusively conducted in Latin America and, therefore, we excluded international trials which may have enrolled participants from this region. Most of the international trials are sponsored by the pharmaceutical industry, which may answer important questions but are less likely to influence the public health of low and middle income countries in the short term.[20] Nonetheless, the Latin America region has also seen good examples of international trials that study generic drugs for prevalent diseases or conditions with direct relevance to the region. For example, Latin America has had an active participation in the MRC CRASH Trial,[21] which evaluated the effect of corticosteroids on head injury, or the CREATE-ECLA trial,[22] which evaluated the effect of the glucose-insulin-potassium (GIK) infusion in patients with myocardial infarction. Another good example is the Magpie trial, which assessed the effects of magnesium sulphate in approximately 10,000 women with pre-eclampsia, including women from Latin America, and resulted in a change of clinical practice.[23] Conducting regional RCTs will also promote the development of skills and infrastructure which will allow Latin America’s researchers to participate in international collaborations addressing these generic but locally relevant questions.

Finally, a further limitation of this report is that we only included RCTs, and it is possible that observational studies carried out in topics such as non communicable diseases and injuries are less likely to be published in the five leading medical journals which may favor RCTs.

### What are the future challenges?

In this paper we only focused on published reports, yet it is widely recognized that only a small proportion of studies reach the publication stage. Unfortunately, at the moment, there is not any source with complete information about RCTs conducted available in Latin America. The World Health Organization is actively promoting an international initiative to develop registers of controlled trials.[24] In Latin America, the Colombian Branch of The Iberoamerican Cochrane Network developed the Latin American Ongoing Clinical Trials Register (LATINREC) which, when fully active, will represent a unique opportunity to obtain a detailed profile of the research conducted in the region.[25] The analysis of this register will confirm or not the gap found in this report and it will enable researchers and policy makers to draw an appropriate research profile of the region. It will be possible to identify duplication of work, inequitable funding of research, neglected diseases and other aspect such as source of funding and quality of design and reporting of clinical trials.[26]

In addition, editorial boards should not only avoid “bias against the diseases of poverty” but, in the context of the epidemiological transition occurring in developing regions, such as Latin America, they should also avoid the bias of what they consider diseases of poverty. This predisposition could partially explain why while Latin America experienced an increase of non-communicable diseases and injuries in the last 20 years, this epidemic was not mirrored by the RCTs published in the five leading medical journals.

## Supporting Information

Appendix S1 (0.03 MB DOC)
