# Supplementary material for: Relation between the Global Burden of Disease and Randomized Clinical Trials Conducted in Latin America Published in the Five Leading Medical Journals
Source: PLoS One. 2008 Feb 27;3(2):e1696. doi: 10.1371/journal.pone.0001696 (PMC2246037; doi:10.1371/journal.pone.0001696)
Supplement: Appendix S1 — (0.03 MB DOC) [file pone.0001696.s001.doc]

**Search strategy in PUBMED**

((clinical trial[pt]) OR (randomized) OR (placebo) OR ("Clinical Trials"[MeSH]) OR (randomly) OR (trial)) AND ((Latin America* [text word] or LatinAmerica* [text word] or South America* [text word] or Caribbea* [text word] or Anguilla [text word] or Antigua and Bermuda [text word] or Argentina [text word] or Aruba[text word] or Bahamas[text word] or Barbados[text word] or Belize[text word] or Bolivia[text word] or Brazil[text word] or Brasil [text word] or British Virgin Islands [text word] or Cayman Islands [text word] or Chile [text word] or Colombia[text word] or Costa Rica [text word] or Cuba [text word] or Dominica[text word] or Dominican Republic [text word] or Ecuador [text word] or El Salvador [text word] or French Guiana[text word] or Grenada[text word] or Guadalupe[text word] or Guatemala [text word] or Guyana [text word] or Haiti [text word] or Honduras[text word] or Jamaica [text word] or Martinique[text word] or Mexico[text word] or Mejico [text word] or Montserrat[text word] or Netherland Antilles [text word] or Nicaragua [text word] or Panama[text word] or Paraguay[text word] or Peru[text word] or Puerto Rico[text word] or St. Kitts and Nevis[text word] or St. Lucia [text word] or St. Vincent and the Grenadines [text word] or Suriname [text word] or Trinidad and Tobago [text word] or Turks and Caicos Islands [text word] or Uruguay [text word] or U.S. Virgin Islands [text word] or Venezuela. [text word]) OR (Latin America* [ad] or South America* [ad] or Caribbea* [ad] or Anguilla [ad] or Antigua and Bermuda [ad] or Argentina [ad] or Aruba[ad] or Bahamas[ad] or Barbados[ad] or Belize[ad] or Bolivia[ad] or Brazil[ad] or Brasil [ad] or British Virgin Islands [ad] or Cayman Islands [ad] or Chile [ad] or Colombia[ad] or Costa Rica [ad] or Cuba [ad] or Dominica[ad] or Dominican Republic [ad] or Ecuador [ad] or El Salvador [ad] or French Guiana[ad] or Grenada[ad] or Guadalupe[ad] or Guatemala [ad] or Guyana [ad] or Haiti [ad] or Honduras[ad] or Jamaica [ad] or Martinique[ad] or Mexico[ad] or Mejico [ad] or Montserrat[ad] or Netherland Antilles [ad] or Nicaragua [ad] or Panama[ad] or Paraguay[ad] or Peru[ad] or Puerto Rico[ad] or St. Kitts and Nevis[ad] or St. Lucia [ad] or St. Vincent and the Grenadines [ad] or Suriname [ad] or Trinidad and Tobago [ad] or Turks and Caicos Islands [ad] or Uruguay [ad] or U.S. Virgin Islands [ad] or Venezuela. [ad])) AND (Lancet[journal] OR BMJ[journal] OR JAMA[journal] OR New England Journal of Medicine[journal] OR Annals of Internal Medicine[journal]) Limits: Publication Date from 1990/01/01 to 2006/12/31
